# Extended release huperzine for the treatment of idiopathic epilepsy in dogs – a Case Report

**DOI:** 10.3389/fvets.2025.1518379

**Published:** 2025-03-13

**Authors:** Kylie L. Grant, Sam N. Long

**Affiliations:** Veterinary Referral Hospital, Melbourne, VIC, Australia

**Keywords:** huperzine, idiopathic epilepsy, anti-epileptic drugs, case series, anticonvulsant

## Abstract

Huperzine is a naturally occurring alkaloid derived from the Chinese clubmoss *Huperzia serrata*. It is a potent acetylcholinesterase inhibitor, among other properties, and has demonstrated protection against induced seizures in a mouse model of Dravet’s syndrome as well as nerve-agent induced seizures and is being explored as a novel anticonvulsant in a human clinical trial for focal impaired awareness seizures. It is also being explored as a treatment for Alzheimer’s, via neuroprotective effects and an ability to ameliorate neuroinflammation. Here we present a case series of 6 dogs with idiopathic epilepsy treated with huperzine to investigate this potential novel anticonvulsant. Despite a 50% drop out rate over the course of the study due to various causes including unexplained death, humane euthanasia and systemic disease, huperzine was generally well tolerated and showed some positive effects on demeanor. This study highlights the need for more research to investigate its efficacy as a novel antiepileptic medication in dogs.

## Introduction

Canine idiopathic epilepsy is a chronic health condition with an estimated prevalence of 0.6–0.75% of the dog population (1, 2) with a much higher incidence in certain dog breeds (3, 4) (18% in a population of Irish Wolfhounds and 33% in a population of Belgian Shepherds from Denmark with common ancestry). Although research continues into non-pharmacological interventions such as dietary alterations (5) and vagal nerve stimulation (6), at present anti-epileptic medications remain the mainstay of treatment for canine epilepsy.

Anti-epileptic drug selection is usually made on the basis of drug factors (e.g., tolerability, dosing frequency, regulatory factors, drug interactions and mechanism of actions), patient factors (e.g., comorbidities, seizure frequency and semiology) and client factors (e.g., drug expense and need for follow-up monitoring).

Approximately 30% of canine idiopathic epileptic patients develop refractory epilepsy which is defined as inadequate seizure control despite adequate serum levels or appropriate drug doses of two or more typically effective anti-epileptic drugs (AEDs). Until recently phenobarbitone and potassium bromide have been considered the primary AED treatment options based on availability, experience and cost. Phenobarbitone has shown a cumulative success rate (ie >50% seizure reduction) of 82% (7) and 73.9% for potassium bromide (7) but many patients are still euthanised due to recurrent seizures or adverse effects of medications (8). In the past two decades other newer generation AEDs have become available in dogs such as imepitoin, levetiracetam and zonisamide with varying levels of evidence to recommend their use either as monotherapy or polytherapy. While more adequately-sized, blinded, randomized controlled trials evaluating the efficacy of all available AEDs for use in canine epilepsy are needed to help fill our gaps in knowledge, new AEDs with improved side effect profile and tolerability for use in dogs are needed.

Huperzine is a naturally occurring sesquiterpene alkaloid derived from the Chinese club moss *Huperzia serrata* (9). It is a potent, highly specific reversible central inhibitor of acetylcholinesterase that can cross the blood brain barrier leading to significantly increased brain acetylcholine levels and has demonstrated anti-epileptic properties in rodents (9). There are two predominant molecular forms of anticholinesterase within the mammalian brain, monomeric and tetrameric. Tetrameric is the predominant form in adult brains and shows partial synaptic localization and thus likely contributes to synaptic transmission (10). Huperzine has the highest potency compared with other acetylcholinesterase inhibitors such as physostigmine and donazepril with a preference for the tetrameric form within the corpus striatum, cortex and hippocampus. One of the proposed anticonvulsant mechanisms of action of huperzine is via enhancement of GABAergic transmission activated by muscarinic receptor activation. Huperzine has also demonstrated anti-inflammatory and neuroprotective effects with proposed mechanisms such as suppression of inflammatory cytokines mediated by nicotinic acetylcholine receptors in the so-called ‘cholinergic anti-inflammatory pathway’ and through reduction of IL-1 beta which is a proposed therapeutic target for drug resistant epilepsy (10). It also reduces Amyloid-beta production, protects cells from Amyloid-β1–42 and Amyloid-β 25–35 and has been shown to improve cognitive improvement in Alzheimer’s patient in clinical trials (11). It is also a weak, dose-dependent NMDA receptor antagonist, although this activity is not likely achieved at tolerable doses in humans (10, 11). Huperzine protects against apoptosis following ischaemia and reperfusion and reduces mitochondrial dysfunction (12). Huperzine has demonstrated anti-seizure effects in mouse models of SCN1A-derived Dravet syndrome and genetic epilepsy with febrile seizures + (GEFS+) (9), nerve agent-induced seizures in the experimental setting (13, 14) and a rat model of kainic acid-induced temporal lobe epilepsy (15). An extended release form of Huperzine A is currently in a phase II clinical trial for focal impaired awareness seizures in people (16).

Huperzine A has been tested in dogs and found to be safe at therapeutic levels (17, 18), before being trailed in a single dog with seizures and behavioral disorders (19). The aim of this prospective case series was to investigate a novel synthetic extended-release form of Huperzine A in a group of canine idiopathic epileptic patients as a potential novel anticonvulsant.

## Method

This single-center prospective case series was designed to investigate the safety and tolerability of a novel synthetic extended-release version of huperzine A (Biscayne Pharmaceuticals, Miami, Florida) in dogs. Inclusion criteria included a body weight of >10 kg and < 40 kg, a seizure frequency of at least 4 generalized tonic clonic seizures per month and a history of either (1) a minimum of 6 months of seizures with normal interictal neurological examination findings, or (2) advanced imaging demonstrating a structurally normal brain (i.e., a diagnosis of idiopathic epilepsy with either Tier I or Tier II diagnosis according to IVETF guidelines). Patients had to have had reactive seizures excluded as a differential, and animals had to have been receiving a usually effective AED at appropriate doses and adequate serum therapeutic levels with unsatisfactory seizure control. Serum therapeutic reference ranges for phenobarbitone and potassium bromide were determined as 65–150 umol/L and 8.8–25 mmol/L, respectively. For patients on levetiracetam, zonisamide and imepitoin, serum levels were not measured since testing and therapeutic drug levels for these drugs are not routinely performed or available in Australia.

Patients were referred to the supervising neurologist (SL) for management of their epilepsy. An initial visit was scheduled in which a full general physical and neurological examination was conducted, assessment of seizure diary and CBC, biochemistry and anti-convulsant serum levels were performed. The clinical trial aims and protocols were discussed with clients and appropriate consent for enrolment performed. Cases were recruited between November 2017 and January 2018.

The treatment phase consisted of the administration of huperzine at a starting dose of 10ug/kg orally every 12 h. Dose escalation occurred after the starting dose treatment period every 2 days to a total of 25ug/kg every 12 h on day 3 and 50ug/kg on day 5. Following day 5 seizure frequency was to be monitored. If seizure frequency remained unacceptable (deemed to be more than 2 seizures per month) the dose was to be increased in 50ug/kg (twice daily) increments until either the seizure frequency was less than once monthly, an adverse event or side effect was encountered or a maximal dose of 300ug/kg was reached. Seizures were recorded using a seizure diary. Revisits with the supervising trial clinician (SL) were to be performed monthly for a total of 5 visits (one visit per month following the commencement of the treatment phase). At each revisit a general physical and neurological examination was performed along with repeat CBC and biochemistry testing. At the final visit a questionnaire was filled out by the client to assess the response to huperzine and any potential side effects or adverse events.

## Results

Six client-owned dogs met the inclusion criteria and were recruited for the study. Breeds included two Australian Kelpies and one of each: Golden Retriever, Staffordshire Terrier, Jack Russell Terrier and Border Collie. Four male dogs were included (1 male entire, 3 desexed) and 1 female desexed. Ages ranged from 2 years to 9 years of age (with a median of 5 years of age). All dogs had normal general physical and neurological examination findings at their initial visit. All dogs were on at least two anti-epileptic medications at the time of treatment phase commencement. Anti-epileptic medications included phenobarbitone, potassium bromide, zonisamide, imepitoin and levetiracetam.

Individual patient summaries are provided below.

### Case 1

**Figure 1:**
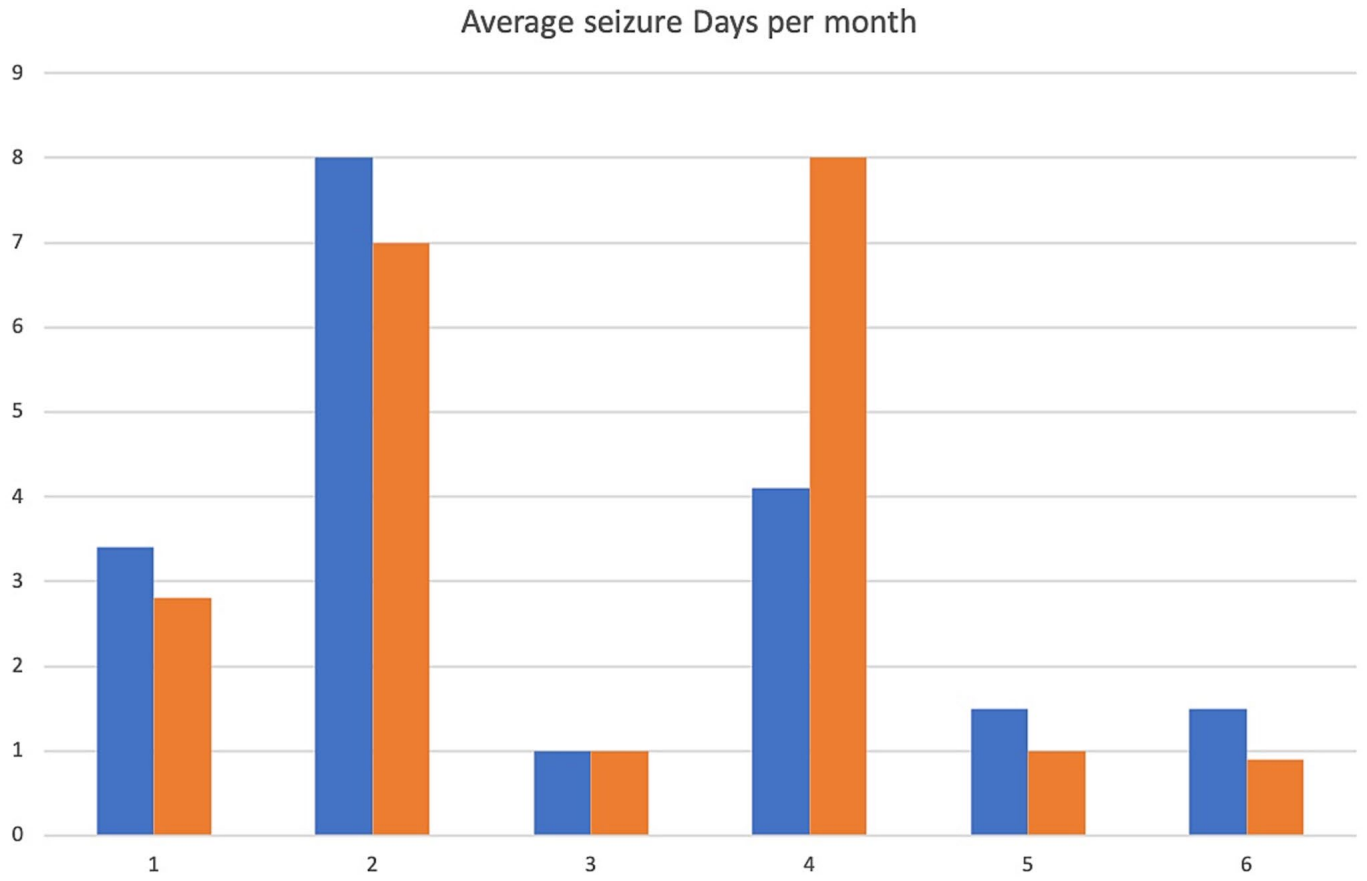
Average seizure days per month. X-axis denotes cases 1–6 and y-axis denotes number of seizure days per month. Pre-treatment values are in blue, intra-trial treatment values in orange.

**Figure 2:**
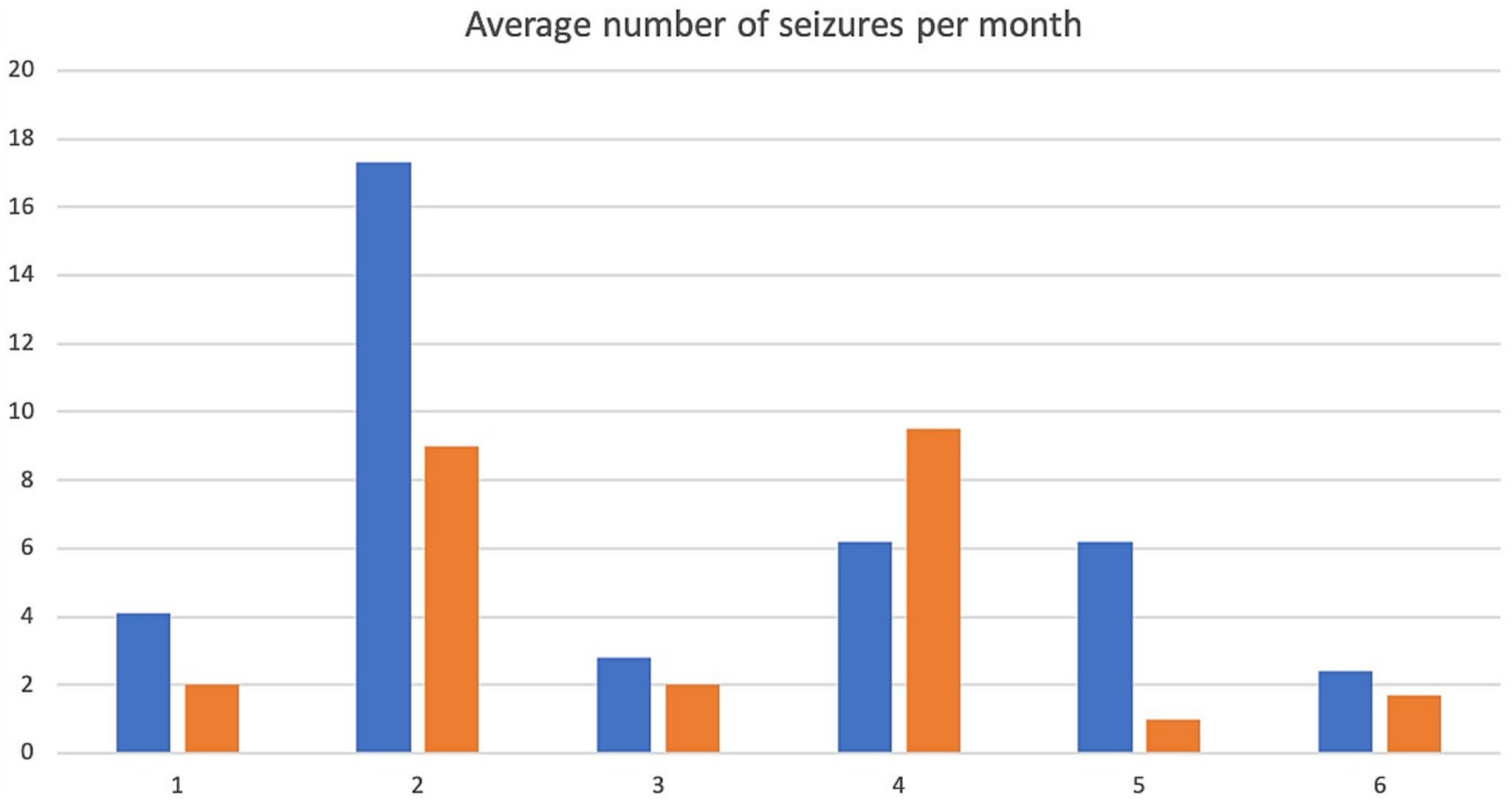
Average number of seizures per month. X-axis denotes cases 1–6 and y-axis denotes number of seizures per month. Pre-treatment values are in blue, intra-trial treatment values are in orange.

**Figure 3:**
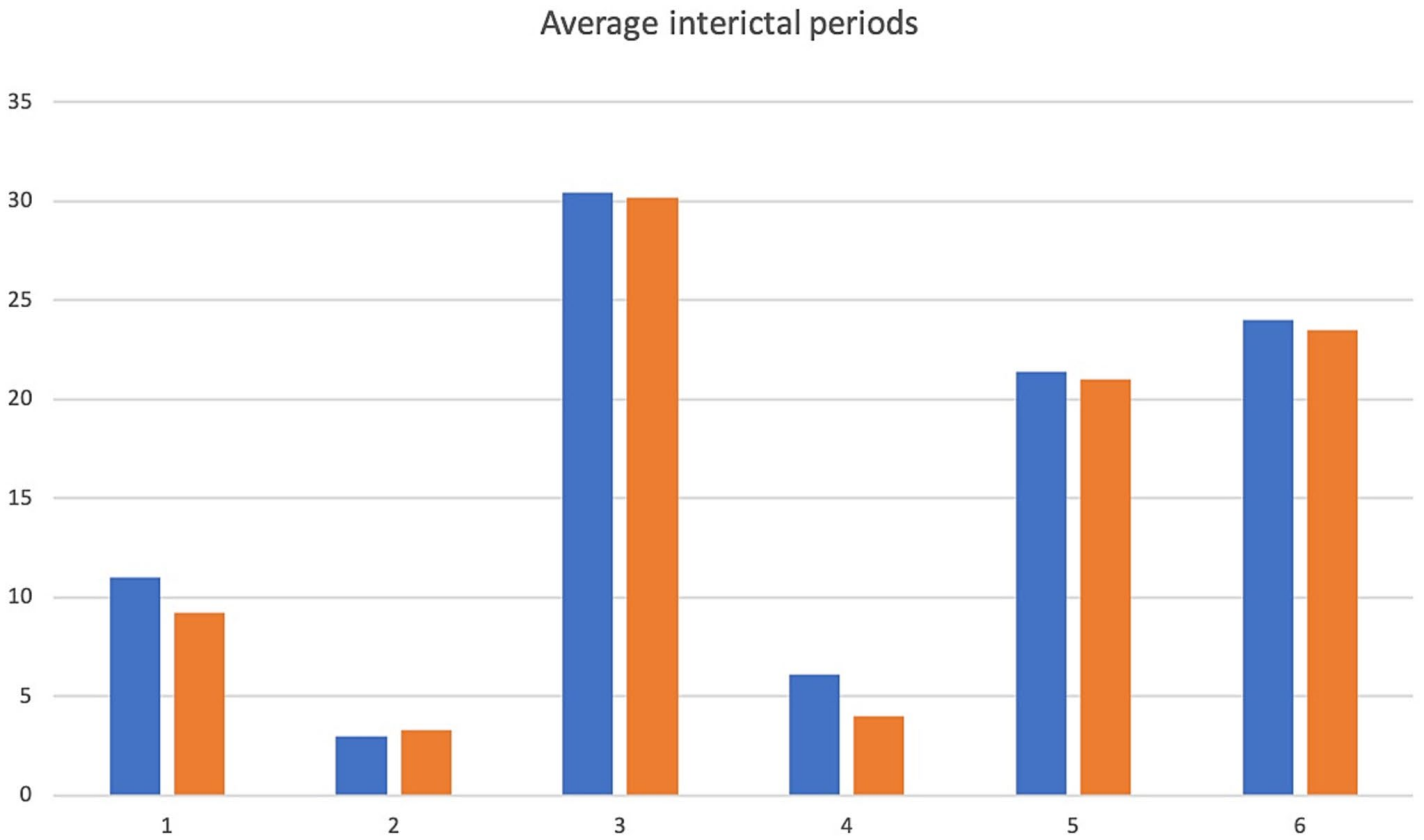
Average interictal period. X-axis denotes cases 1–6 and y-axis denotes inter-ictal periods in days. Pre-treatment values in blue, intra-trial treatment values are in orange.

A 5.5 yr. MN Kelpie was recruited in November 2017 with a history of idiopathic epilepsy with the first seizure occurring at 4 years of age. The patient experienced predominantly generalized tonic clonic seizures but also experienced occasional focal seizures characterized by facial twitching which occurred as either single or cluster events every 1–2 weeks. The patient had been started on phenobarbitone, potassium bromide and imepitoin at appropriate doses and serum drug levels for phenobarbitone and potassium bromide were within therapeutic reference ranges. The patient had a mild hyperchloraemia and decreased anion gap, suspected to be associated with bromide administration, as well a mild increased ALP which was suspected to be associated with phenobarbitone and/or potassium bromide therapy (see Table 1) on initial serum biochemistry and hematology testing. Average seizure days per month recorded for 7 months prior to study commencement was 3.4 (mean 4). Average number of seizures per month recorded prior to study commencement was 4.1 and average interictal period was 11 days (See Figures 1–3).

**Table 1:** Biochemical/hematological changes.

Case number	Pre-treatment abnormal values	Intra-treatment abnormal values
1	Chloride-127 mmol/L Anion gap-5.4 mmol/L ALP-151 IU/L	Week 5: - ALT 187 IU/L ALP 181 IU/L Chloride 126 mmol/L Anion gap 7 mmol/L Cholesterol 3.31 mmol/L Week 10: - ALP 251 IU/L Chloride 123 mmol/L Anion gap 5 mmol/L Week 20: WBC-26.4 × 10^9^/L Neutrophils-18.0 × 10^9^/L Monocytes-2.1× 10^9^/L Lymphocytes-0.79× 10^9^/L HCT-0.33 L/L Albumin-18 g/L ALT-312 IU/L AST-130 IU/L ALP-520 IU/L Chloride-121 mmol/L Anion gap-6 mmol/L Bile acid tolerance testing - pre-prandial 74umol/L, post-prandial 55umol/L Week 24 (after disenrollment from trial): ALP 164 IU/L Anion gap 3 mmol/L
2	WBC-5.6× 10^9^/L Lymphocytes-0.9× 10^9^/L Urea-10.2 mmol/L ALT-93 IU/L ALP-182 IU/L Cholesterol-13.6 mmol/L	Week 4: WBC-3.7 × 10^9^/L Lymphocytes-0.8×10^9^/L Neutrophils-2.6×10^9^/L ALP-183 IU/L Cholesterol 13.9 mmol/L
3	Chloride-122 mmol/L Anion gap-9 mmol/L ALT-98 IU/L	Week 12: Chloride-125 mmol/L Anion gap 6 mmol/L Potassium 3.7 mmol/L Week 16: - ALT 99 IU/L Chloride 124 mmol/L Anion gap 8 mmol/L Week 20: - ALT 146 IU/L ALP 159 IU/L CK 568 IU/L Chloride 121 mmol/L
4	Hematocrit- ALP- 274 IU/L Cholesterol-8.7 mmol/L	Week 3: ALP 274 IU/L Cholesterol 8.7 mmol/L Week 17: ALP 344 IU/L Week 25: ALP 405 IU/L Cholesterol 9.2 mmol/L Globulins-37 g/L
5	ALT-546 IU/L ALP-1164 IU/L Albumin-19 g/L	Week 4: ALT-299 IU/L ALP-772 IU/L AST-102 IU/L Albumin-20 g/L Cholesterol 2.83 mmol/L
6	ALP-159 IU/L Cholesterol-12.95 mmol/L	Week 3: Cholesterol-9.3 IU/L Week 10: Cholesterol-11.2 mmol/L Creatinine-138 IU/L Week 14: Cholesterol-13.5 mmol/L Week 19: Cholesterol-11.3 mmol/L Creatinine-147umol/L Week 28: Cholesterol-12.4 mmol/L

The patient was started on 10ug/kg PO q12 and underwent dose escalation uneventfully as per the treatment protocol to 50ug/kg PO q12. He experienced 1 episode of vomiting during week 5 of treatment however no treatment was required, huperzine was continued and the vomiting self-resolved. The dose was increased at week 10 to 100ug/kg PO q12 due to ongoing inadequate seizure control and again to 200ug/kg PO q12 at week 19.

Repeat CBC and biochemistry panels were performed at week 5 which identified mild increases in ALT and ALP, mild hyperchloraemia with decreased anion gap and mild hypocholesterolaemia. Phenobarbitone and bromide levels were not reassessed. CBC and biochemistry panels at week 10 showed a further mild increase in ALP, normal ALT values and mild hyperchloraemia and decreased anion gap.

The patient presented to the emergency service during week 20 of treatment after experiencing a single seizure followed by progressive tetraparesis, dull mentation and pyrexia. On repeat CBC and biochemistry testing a mild leukocytosis due to a mild neutrophilia with a left shift, monocytosis and lymphocytosis and mild non-regenerative anemia, mild hypoalbuminaemia, mild increased ALT, AST and ALP as well as mild hyperchloraemia and decreased anion gap were evident. Bile acid tolerance testing identified elevated pre-and post-prandial acid levels. An abdominal ultrasound was performed which was unremarkable however fine needle aspirates of the liver were consistent with bacterial cholangiohepatitis with evidence of abscessation. Phenobarbitone doses were reduced and weaned due to concerns over hepatic insuffiency, antibiotic therapy was commenced and the patient demonstrated marked clinical improvement as well as resolution of his hematological and biochemical changes 4 weeks later aside from a mildly elevated ALP and mild decreased anion gap. He was disenrolled from the study although continued on huperzine until he was euthanised 7 months later due to ongoing poor seizure control. Average seizure days for the 5 months of trial enrolment was 2.8, average number of seizures per month was 2 and average interictal period was 9.2 days (see Figures 1–3).

### Case 2

A 9 yr. Border Collie was recruited to the trial in January 2018 with a history of seizures from 8 years of age. CT of the brain which was unremarkable and CSF cytologically normal. The patient experienced cluster generalized tonic clonic seizures every 1–2 weeks with cluster of up to 15 seizures with occasional focal seizures (manifesting as facial twitching) and was currently receiving phenobarbitone, imepitoin and zonisamide at appropriate dose rates and measurable serum levels were within therapeutic reference ranges. CBC and biochemistry panels performed at recruitment identified a marginal leukopaenia due to a marginal lymphopaenia, mild increased urea, as well as mild increased ALT, ALP and mild hypercholesterolaemia which were attributable to phenobarbitone administration (see Table 1).

Average seizure days per month for 3 months recorded prior to trial commencement was 8, average number of seizures per month was 17.3 and average interictal period was 3 days (see Figures 1–3).

Huperzine was started at 10ug/kg PO q12 and escalated to 50ug/kg PO q12 as per the treatment protocol. The dose was increased to 100ug/kg PO q12 at week 6 due to ongoing inadequate seizure control. All dose escalations occurred uneventfully aside from one episode of vomiting at week 4 which self-resolved. Repeat CBC and biochemistry testing showed mild leukopaenia due to mild neutropaenia and lymphopaenia as well as mild increased ALP and hypercholesterolaemia.

Due to increasing difficulties with tableting leading to missed doses as well as ongoing poor seizure control his owners elected for euthanasia at week 10. Average seizure days per month for the 10 weeks of enrolment was 7, average number of seizures per month was 9 and average interictal period was 3.3 days (see Figures 1–3).

### Case 3

A 7 yr. MN Jack Russell Terrier was recruited to the trial in November 2017 with a history of seizures from 5.5 years of age. No abnormalities were identified on CT scan performed previously. The patient experienced generalized tonic clonic seizures usually in clusters, although he occasionally experienced isolated events, every 1–3 weeks. However, for the 10 weeks prior to enrolment he was seizure-free (after a bromide dose adjustment) which was followed by a severe cluster event prior to trial commencement. Current anti-epileptic medications were phenobarbitone and potassium bromide at appropriate doses and with serum levels within therapeutic reference range. On initial CBC and biochemistry testing, a mild hyperchloraemia and decreased anion gap, attributable to bromide administration, and mild increased ALT likely associated with phenobarbitone and/ or bromide administration (see Table 1) were noted.

Average seizure days per month for 6 months prior to trial commencement was 1, average number of seizures per month was 2.8 and average interictal period was 30.4 days (see Figures 1–3).

The patient was started on 10ug/kg PO q12 and escalated to 50ug/kg PO q12 uneventfully. The owners ran out of medications for 3 days during week 5 of treatment so the low dose was restarted with a second dose escalation to 50ug/kg Po q12 which also occurred without issue. The dose was increased to 100ug/kg PO q12 at week 14 due to inadequate seizure control.

Repeat CBC and biochemistry panels were performed at week 4 of treatment which identified mild hypokalaemia and mild hyperchloraemia with decreased anion gap. On CBC and biochemistry testing at week 12 of treatment, a mild hypochloraemia, decreased anion gap and marginal hypokalaemia were noted. Week 16 CBC and biochemistry panels showed mildly increased ALT and mild hyperchloraemia and decreased anion gap and week 20 panels demonstrated a mildly increased ALT, mildly increased ALP, mildly increased CK and mild hyperchloraemia.

The patient completed the clinical trial. Average seizure days per month during the trial was 1, average number of seizures per month was 2 and average interictal period was 30.2 days (see Figures 1–3).

### Case 4

A 2 yr. FN Staffordshire Bull Terrier was recruited to the trial in November 2017 with a history of generalized tonic clonic seizures from 1 year of age. The patient experienced single or isolated generalized tonic clonic seizures every 3–4 days. The patient was receiving phenobarbitone and levetiracetam at the time of enrolment. A mildly increased hematocrit, which was interpreted as haemoconcentration at the time of sample collection, mildly increased ALP and mild hypercholesterolaemia (see Table 1) were evident on initial CBC and biochemistry testing. Serum phenobarbitone levels were within therapeutic reference range. Average seizure days per month for 5 months prior to huperzine commencement was 4.2, average number of seizures per month was 6.2 and average interictal period was 6.1 days (see Figures 1–3).

The patient was started on 10ug/kg PO q12 huperzine and underwent dose escalation to 50ug/kg and subsequently to 100ug/kg uneventfully. Mild ongoing increases in ALP and cholesterol were apparent on repeat CBC and biochemistry panels performed at week 3 (at 100ug/kg dose). An initial perceived improvement in seizure control was noted at this dose however the dose was once again increased at week 10 to 200ug/kg due to ongoing poor seizure control. Repeat CBC and biochemistry panels identified a mild increase in ALP and mildly increased hematocrit.

At week 17 her dose was increased to 300ug/kg PO q12 due to ongoing inadequate seizure management. Repeat CBC and biochemistry panels showed a mildly increased ALP.

At her exit examination at week 25, on repeat CBC and biochemistry, mildly increased ALP, mild hypercholesterolaemia and mild hyperglobulinaemia were noted.

Average seizure days per month during the trial was 8, average number of seizures per month was 9.5 and average interictal period was 4 days (see Figures 1–3).

### Case 5

An 8 yr. MN Kelpie was recruited to the trial in November 2017 with a history of generalized tonic clonic seizures since the patient was 6 years of age. His seizures usually occurred in clusters approximately every 3 weeks. He was receiving phenobarbitone, levetiracetam and imepitoin. Mildly increased ALT, moderate elevations in ALP and mild hypoalbuminaemia (see Table 1) were evident on baseline CBC and biochemistry panels. Serum phenobarbitone levels were appropriate. The patient was started on huperzine 10ug/kg PO q12 with dose escalation to 50ug/kg PO q12 without reported incident. Repeat CBC and biochemistry panels were performed at week 4 which detected ongoing mild hypoalbuminaemia and mildly increased ALT, moderately increased ALP, mildly increased AST and mild hypocholesterolaemia. Average seizure days per month for 6 months prior to trial commencement was 1.5 and average number of seizures per month was 6.2 (see Figure 1).

The dog was unfortunately found dead in the owner’s backyard on week 8 of the trial. There was evidence of seizure activity surrounding his body (saliva, urine and faces) however a post-mortem was declined by the owner. Average number of seizure days for the 2 months of the trial was 1, average seizures per month was 1 and average interictal period was 21 days (see Figures 1–3).

### Case 6

A 4 yr. MN Golden Retriever was enrolled in November 2017 with a 6-month history of GTC seizures. He experienced a combination of isolated and cluster seizures usually every 1–3 weeks. He was receiving imepitoin and levetiracetam, he had been initially started on phenobarbitone at the onset of seizures however this was weaned to imepitoin after 2 months due to adverse side effects of phenobarbitone. Mildly increased ALP and hypercholesterolaemia (see Table 1) were apparent on baseline CBC and biochemistry panels. Average seizure days per month in the recorded in the 7 months prior to trial start was 1.5, average number of seizures per month was 2.4 and average interictal period was 24 days (see Figures 1–3).

Huperzine was started at 10ug/kg PO q12 and escalated to 50ug/kg PO q12 with no reported adverse effects. The dose was subsequently increased to 100ug/kg PO q12 at week 14 due to inadequate seizure control.

Repeat CBC and biochemistry panels at week 3 of the trial identified a mild hypercholesterolaemia. CBC and biochemistry panels were performed at week 10 demonstrated mild hypercholesterolaemia and mildly increased creatinine. CBC and biochemistry panels performed at week 14 detected ongoing mild hypercholesterolaemia, week 19 mild hypercholesterolaemia and mildly increased creatinine and at exit exam at week 28 mild hypercholesterolaemia.

Average number of seizure days per month during the trial was 0.87, average number of seizures per month was 1.7 and average interictal period was 23.5 days (see Figures 1–3).

## Discussion

We report a case series of 6 dogs with refractory epilepsy treated with a novel anticonvulsant, huperzine. Huperzine generally appeared well-tolerated with cases 1 and 2 reporting transient vomiting during dose escalation periods which resolved without treatment. Case 1 was diagnosed with bacterial cholangiohepatitis in week 20 and was disenrolled from the clinical trial however continued on huperzine at previous doses without adverse effects noted. Case 5 was found dead in the backyard during week 8 of the trial. Unfortunately, his owners did not consent to a post mortem which would have been ideal to exclude an adverse event associated with huperzine administration. However, his death was thought to be secondary to an episode of status epilepticus given the circumstances in which he was found with evidence of recent seizure activity.

No hematological changes were noted during treatment, relative to pre-treatment values for any of the dogs within the trial. Case 1 recorded a new increase in ALT at week 5 which resolved at week 10 as well as a further mild increase in ALP prior to marked biochemical changes notes at week 20 secondary to bacterial cholangiohepatitis and subsequent liver dysfunction. These biochemical changes resolved, aside from a mild elevation in ALP and mild decreased anion gap upon initiation of antibiotic therapy and reduction of phenobarbitone dose. Case 3 recorded a further mild increase in ALT relative to pre-treatment values as well as a novel increase in ALP and CK at week 20. A further mild increase in ALP in case 4 was noted compared to pre-treatment value at weeks 17 and 25. Case 5 recorded a mild reduction in previously elevated ALP and ALT values as well as a mild increased in AST and hypocholesterolaemia. No investigation was performed to determine the underlying cause of mild hypoalbuminaemia, however this remained static during treatment. The mild hypercholesterolaemia noted in case 6 remained static throughout the trial and mild elevations in creatinine were noted at week 10 and week 19 but not present at final testing in week 28.

Huperzine A has previously been demonstrated to have rapid and near complete oral absorption after single oral administration with mean oral bioavailability of 94.4% as measured in a case series of 5 healthy Beagles (18). There are no established huperzine serum therapeutic levels for humans [interestingly huperzine seems to have good controlled release properties via transdermal route in dogs (17)]. In people, huperzine A is excreted largely unchanged within the kidneys rather than undergoing extensive hepatic metabolism and thus is unlikely to cause significant drug:drug interactions when used in conjunction with medications metabolized by the cytochrome p450 isoenzyme system, such as phenobarbitone (20).

The average number of seizure days per month before and during treatment were recorded as well as average number of seizures per month and average interictal periods before and during treatment and are displayed in Figures 1–3. We have not attempted to look for statistically significant changes in these parameters due to the low case numbers involved and the high dropout rate. Unfortunately, external factors dictated the recruitment phase of the trial and a longer period of enrolment would certainly have resulted in a larger case series. Although case 1 continued on huperzine after disenrollment from the trial, his owners continued to keep a seizure diary and thus this is included in the results section and figures.

Patients 1, 2, 5 and 6 demonstrated a decrease in seizure days per month and patients 1, 2, 3, 5 and 6 a decrease in average seizures per month (Figures 1, 2) although this was less than a 50% improvement in seizure number or reduction in interictal periods. Measurement for statistical significance was not attempted.

Interestingly, the owners of cases 3 and 6 also reported a dramatic improvement in quality of life due to increased energy levels, decreased restlessness and increased responsiveness. Both of these owners elected to continue huperzine after trial cessation as a result of this perceived improvement in quality of life. There is some evidence to suggest that huperzine may improve cognitive function, daily living activity and global clinical assessment in human Alzheimer patients (21) and is licensed as an anti-Alzheimer drug in China. Its use in traditional Chinese medicine has also been to improve mental sharpness, memory and acuity (11). It is possible that the reported improvement in quality of life assessments in these two canine epileptic patients may be associated with huperzine’s apparent neuroprotective effects and this warrants further investigation for its use as an anticonvulsant. One of the most common complaints of the owners of dogs with epilepsy is the decrease in quality of life associated with AED side effects, sedation, ataxia and increased thirst and (22). An anticonvulsant that does not induce these side effects, and which could even improve mental function, would be an extremely attractive option for many owners.

There are several limitations to this study. Obviously sample size and the high dropout rate were issues that precluded any meaningful assessment of the efficacy of huperzine as an anticonvulsant. A further, larger, randomized and blinded clinical trial is certainly warranted for this purpose. In addition, measurement of serum levels of huperzine and definitive confirmation of its lack of interaction with other anticonvulsants with serial serum level monitoring is important. Dose escalation also occurred at random intervals based on seizure frequency and owner updates. Another limitation is the lack of advanced imaging in all cases. Cases 2 and 3 had CT of the brain performed as MRI was not readily available at the study center at the time. Advanced imaging with MRI of the brain would have been ideal for all cases. However, idiopathic epilepsy was deemed the most likely diagnosis in the other cases based on signalment, history and normal interictal exams and thus we felt a Tier I IVETF diagnosis of idiopathic epilepsy had been reasonably established.

One other limitation is the lack of a post mortem confirming the cause of death of patient 5. However, his owners found him surrounded by evidence of recent seizure activity and so the most plausible explanation would be terminal episode of status epilepticus or possibly sudden unexpected death in epilepsy (23).

## Summary

In this small population of refractory canine epileptic patients huperzine appeared well tolerated. Further studies are required to investigate the potential anti-convulsive activity of huperzine in a larger group of patients.

## Data Availability

The raw data supporting the conclusions of this article will be made available by the authors, without undue reservation.
